# Transformative strategies for enhancing women’s resilience to climate change: A policy perspective for low- and middle-income countries

**DOI:** 10.1177/17455057241302032

**Published:** 2024-11-22

**Authors:** Mudassar Aziz, Gulnaz Anjum

**Affiliations:** Department of Psychology, University of Oslo, Oslo, Norway

**Keywords:** climate change, women’s health, resilience, climate policy, low- and middle-income countries

## Abstract

This policy paper advocates for a transformative strategy to address the disproportionate impact of climate change on women in low- and middle-income countries (LMICs), emphasizing the need to integrate gender considerations into climate resilience initiatives. Recognizing the multifaceted nature of women’s vulnerabilities, the paper calls for the dismantling of discriminatory socio-cultural norms and the enhancement of women’s capacities through digital health literacy, political empowerment, and the protection of sexual and reproductive health rights. Focusing on the health implications of climate change, particularly for pregnant women and newborns, the paper promotes a multi-sectoral approach that strengthens health systems and encourages community-based interventions. It underscores the importance of incorporating gender perspectives into climate adaptation and mitigation strategies, advocating for tailored health services in LMICs, and promoting women’s active involvement in climate-related decision-making processes. The methodology involves a qualitative, expert, and narrative synthesis of existing literature and policy analysis. The paper synthesizes existing research and policy recommendations to argue for a comprehensive policy framework and backs it with case studies from LMICs. This framework recognizes the complex interplay between women’s vulnerabilities and climate change, advocating for women’s empowerment as central to climate resilience efforts in LMICs. By integrating gender perspectives, enhancing health services for women, and fostering international collaboration, it proposes a holistic approach to mitigate the adverse effects of climate change on women’s health and well-being. This approach not only acknowledges the specific challenges faced by women but also leverages their unique insights and experiences, positioning them as pivotal contributors to global climate resilience and sustainability efforts.

## Plain language summary

This paper looks at how climate change affects women more than men. • It explains that women face higher health risks due to their social roles and less access to resources. • The paper argues that climate strategies need to include a focus on gender differences. • It suggests improving women’s digital health skills, political power, and access to reproductive health services. • The paper calls for stronger healthcare systems and more community support to help women adapt to climate changes. • It also stresses that including women in climate decisions and working together globally can better protect women and build stronger, more sustainable communities.

## Introduction

Climate change is a critical global challenge that disproportionately affects women’s health, particularly in low- and middle-income countries (LMICs). This policy paper synthesizes the latest research to propose policy pathways for empowering women’s health resilience in the face of climate change. The paper is focused generally on the health and well-being of the women in the Global South,^1^ and more specifically on women in LMICs.

Climate change intensifies health risks, with women in LMICs bearing the brunt of these impacts. Key aspects of this issue include women’s heightened exposure to climate risks, the disproportional adverse effects on their health, the factors contributing to their vulnerability, and the strategies for response^2^ Women in LMICs are particularly vulnerable to the health hazards posed by climate change, a vulnerability exacerbated by existing gender disparities and socio-cultural constraints. In addition to the direct effects of climate change (heatwaves and floods), this susceptibility is often due to factors such as prescribed societal roles, economic dependency, and limited access to healthcare and education.^3,4^ The ramifications of climate change, including nutritional deficiencies, mental health issues, and pregnancy-related complications, are thus more pronounced for women, further strained by often inadequate healthcare infrastructures in these regions.^2^ Climate change adversely affects essential life-supporting systems, leading to shifts in disease patterns and disproportionately impacting the most vulnerable groups, including women, the elderly, and newborns.^5^

Climate-induced disasters such as hurricanes and floods exacerbate public health crises, resulting in famine, food insecurity, and malnutrition, with women and children in economically disadvantaged areas being especially at risk.^6^ Additionally, entrenched gender norms in LMICs contribute to discriminatory practices, further exposing women to natural hazards and a host of socio-economic and health challenges, including psychological stress, chronic fatigue, and limited access to healthcare.^7^ Moreover, the direct impact of climate change on women’s mental health, manifesting as depression, post-traumatic stress disorder, and other neuropsychiatric symptoms, cannot be overlooked.^8^

The psychological impacts of climate change on women in LMICs can be profound, often creating a state of being “limbo.” This term, explored in depth by Anjum, Aziz, and Hamid,^9^ describes the precarious mental state experienced by individuals affected by prolonged uncertainty and instability due to severe external stressors such as climate changes and war. Their research on the physical and mental health of civilians, asylum seekers, and refugees highlights how continuous exposure to traumatic events, coupled with the ongoing uncertainty about their future, leads to significant psychological distress. Similarly, women in LMICs facing climate-induced adversities may experience a similar “limbo” state, characterized by heightened physical distress, mental stress, anxiety, and a sense of helplessness. This comparison underscores the need for targeted mental health interventions and resilience-building strategies that consider the multifaceted vulnerabilities of women in these regions. These psychological threat multipliers resulting from climate shocks and slow onset events are likely to create a situation of being in limbo where women face ongoing uncertainty and instability due to the compounded effects of climate change and socio-economic challenges.^9^

Addressing these challenges necessitates a comprehensive policy response, which includes the incorporation of gender perspectives into climate adaptation and mitigation initiatives, the fortification of health systems to cater specifically to women’s health needs in LMICs, and the promotion of women’s involvement in climate-related decision-making. The investment in gender-specific data and research is critical for a nuanced understanding of climate change’s impacts on women. Furthermore, international cooperation and support are indispensable in aiding LMICs to implement gender-responsive climate policies effectively. Tackling the disproportionate effects of climate change on women’s health in LMICs demands a multi-dimensional policy strategy. This strategy should emphasize the integration of gender perspectives in climate policies, the enhancement of health services tailored for women, the empowerment of women in decision-making roles, targeted research initiatives, and the promotion of international collaboration and assistance. Such concerted efforts are essential for effectively mitigating the challenges climate change presents to women’s health in these regions.

### Current study

The current research is rooted in the urgent need to understand the disproportionate impact of climate change on women, particularly in LMICs. Climate change exacerbates existing gender inequalities and creates new vulnerabilities for women due to their socio-economic status, cultural roles, and limited access to resources. Women often bear the brunt of climate-induced health risks, which include increased incidence of heat-related illnesses, vector-borne diseases, and complications during pregnancy.^2^ The heightened vulnerability of women necessitates a focused and gender-responsive approach to climate resilience that has been largely absent in current policies.^3^

The rationale for this paper lies in the urgent need to integrate gender perspectives into climate resilience strategies. Women in LMICs face unique vulnerabilities due to socio-cultural norms, inadequate health services, limited political participation, and lack of access to information and resources. Addressing these challenges requires a comprehensive approach that includes dismantling discriminatory norms, enhancing health services, promoting digital literacy, and empowering women in decision-making processes. By synthesizing existing research and policy recommendations, this paper aims to provide a robust framework for developing gender-responsive climate resilience policies that not only protect women from the adverse effects of climate change but also empower them to be active contributors to sustainable and equitable solutions.

### Research objectives

1. Analyze the gender-specific impacts of climate change on women’s health in LMICs.
2. Evaluate existing climate policies and identify gaps in addressing women’s resilience.
3. Propose strategies to enhance women’s resilience through digital health literacy, political empowerment, and reproductive health rights.
4. Advocate for multi-sectoral and international collaboration in integrating gender perspectives into climate adaptation and mitigation efforts.

## Method

This policy paper employs a qualitative, expert, and narrative synthesis approach to examine and compile existing literature on transformative strategies for enhancing women’s resilience to climate change from a policy perspective. The focus is on integrating gender considerations into climate resilience initiatives, particularly within LMICs. The methodology involves a comprehensive literature review, analysis of existing policies, and synthesis of research findings to propose a policy framework addressing the intersection of gender, health, and climate resilience.

### Literature search strategy

The literature search was conducted using Web of Science, and Google Scholar. The search terms used included combinations of the following keywords: “women,” “climate change,” “resilience,” “health,” “gender,” “policy,” “low- and middle-income countries,” and “maternal health.” Boolean operators (AND, OR) were used to refine the search and ensure comprehensive retrieval of relevant studies. This search was first discussed with Climate change and gender experts, one from the Global North and the other from the Global South.

### Inclusion and exclusion criteria

The selection of studies for this review adhered to specific inclusion and exclusion criteria to ensure the relevance and quality of the data. The inclusion criteria encompassed studies published between 2000 and 2023, ensuring that the data were current and pertinent. Only peer-reviewed articles, policy reports, and review papers were considered to maintain high academic standards. The focus was on studies that examined the impact of climate change on women’s health in LMICs and those addressing gender-specific vulnerabilities and adaptation strategies. Additionally, only articles available in English were included to facilitate comprehensive analysis and synthesis. Conversely, studies were excluded if they did not address the intersection of gender and climate resilience. Articles that focused solely on high-income countries were omitted to maintain the review’s relevance to LMICs. Non-peer-reviewed articles, opinion pieces, and editorial comments were also excluded to ensure the integrity and reliability of the sources.

### Analytical framework

The analytical framework underpinning this review is deeply rooted in feminist theory and the concept of intersectionality, recognizing the intricate and dynamic interplay of gender, socio-economic status, cultural norms, and environmental factors in shaping women’s experiences and responses to climate change. This approach acknowledges that women’s vulnerabilities and resilience strategies are not unilateral but are influenced by a combination of intersecting identities and social categories that collectively impact their capacity to adapt to and mitigate the effects of climate change.

Feminist theory provides a critical lens for understanding how gender power dynamics shape women’s experiences of climate change. It emphasizes the importance of addressing structural inequalities and power imbalances that marginalize women and limit their access to resources, opportunities, and decision-making processes.^10,11^ Feminist theory argues that climate policies and resilience strategies must be inclusive and address the specific needs and contributions of women to be effective.^10^ Contributions from feminist scholars in the Global South, such as Naila Kabeer and Vandana Shiva, emphasize the intersection of gender with other social categories like class and ethnicity, highlighting how these intersecting factors create unique challenges for women in the context of climate change.^12,13^ Additionally, research exploring the ethics of care in communitarian societies underscores how cultural norms influence gender roles and ethical decision-making, further illustrating the need for culturally sensitive approaches in policymaking.^14^

## Results

This section presents the results and discussion based on the analysis within the analytical framework grounded in feminist theory and intersectionality. The selected papers were examined through the lens of three core themes: health impacts, women’s empowerment and capacity building, and policy integration. This comprehensive approach not only underscores the multifaceted nature of women’s resilience to climate change but also advocates for transformative policies that address the root causes of gender disparities in climate vulnerability. By emphasizing inclusive, equitable, and gender-responsive climate strategies, this analysis aims to empower women and enhance their capacity to contribute to sustainable solutions.

Selected papers in this research were based on the analysis within the framework discussed above. These were structured around three core themes: health impacts; women’s empowerment and capacity building, and policy integration. By grounding the analysis in feminist theory and intersectionality, this framework not only highlights the multifaceted nature of women’s resilience to climate change but also advocates for transformative policies that address the root causes of gender disparities in climate vulnerability. It emphasizes the need for inclusive, equitable, and gender-responsive climate strategies that empower women and enhance their capacity to contribute to sustainable solutions.

### Health impacts

This theme examines the direct and indirect effects of climate change on women’s health, including maternal and newborn health. Climate-related stressors such as heatwaves, flooding, and vector-borne diseases disproportionately affect women, exacerbating health disparities.^2,6^ Intersectional analysis reveals that women in LMICs, especially those from marginalized groups, face higher risks due to inadequate healthcare infrastructure, socio-economic constraints, and cultural barriers.^15–17^

#### Maternal and newborn health policy

Climate change presents immediate and long-term challenges, especially to vulnerable groups such as pregnant women and newborns, through climate shocks, phenomena like heat stress, and floods. Addressing these challenges requires a holistic, multi-sectoral strategy focused on fortifying health systems. This includes enhancing the resilience of infrastructure to withstand extreme climate conditions and offering specialized education to both healthcare professionals and the public regarding the climate-related risks to maternal and neonatal health.^18^

The formulation and execution of specific climate adaptation and mitigation strategies are imperative. Such strategies should guarantee the availability of clean water, sufficient nutrition, and secure shelter during severe climate events, elements crucial for the health and well-being of pregnant women and newborns.^19^ Additionally, the establishment of weather warning and weather surveillance systems to track the impact of climate change on maternal and neonatal health, coupled with investment in research to comprehend distinct risks and formulate appropriate interventions, is essential.^20^

Community-based interventions can significantly bolster the resilience of pregnant women and newborns to the impacts of climate change. Local health education initiatives and community support networks are instrumental in this regard.^21^ Moreover, mitigating environmental exposures like air pollution and extreme temperatures, which disproportionately affect pregnant women and newborns, is crucial. Urban planning and nature-based solutions can offer effective mitigation strategies.^22^ Ensuring the security of food and water, particularly during climate-induced disasters, is another focal area. Policy measures aimed at sustaining stable food supplies and ensuring the provision of safe drinking water are vital for the health of pregnant women and newborns.^23^ The involvement of obstetricians and gynecologists in climate adaptation plans is critical to advocate for and shape policies that safeguard pregnant women and newborns from the detrimental impacts of climate change. Their expertise is indispensable in influencing health policies and public awareness initiatives.^24^ The mental health repercussions of climate change on pregnant women, including anxiety, depression, and stress-related disorders, warrant serious attention. Integrating mental health support into prenatal and postnatal care is paramount to ensure the overall well-being of pregnant women and their newborns.^8^

### Empowerment and capacity building

This theme explores strategies to enhance women’s resilience through education, political empowerment, and community-based interventions. Increasing digital health literacy, fostering women’s leadership in climate-related decision-making, and supporting community initiatives are critical to bolstering women’s adaptive capacities.^3,4^ Intersectional approaches ensure that empowerment efforts are inclusive and address the specific needs of women from diverse backgrounds.^25^

#### Gender equality in climate policy

Progress in climate change mitigation and adaptation is frequently impeded by entrenched gender disparities, often perpetuated by traditional perceptions of women’s roles and characteristics. A nuanced, multidimensional strategy is imperative to surmount these obstacles and foster enhanced gender equality in climate policy.^26^

In their 2020 study, Anwar et al. explored the intersectionality of social, gender, and institutional dynamics, shedding light on the disproportionate burdens borne by women due to climate-induced water scarcity in Karachi. The research highlights how, amidst the deteriorating water infrastructure and governance, women are compelled to uphold household responsibilities, including water management, even during severe droughts. The depletion of aquifers, a primary source of drinking water, is exacerbated by climate change and inadequate water governance, critically undermining household water security. Women, bound by the cultural practice of purdah, which dictates seclusion in private spaces to uphold family honor, face intensified hardships. The dwindling water supply not only amplifies their domestic burdens but also subjects them to “psychological violence,” as they struggle to fulfill their caretaking roles amidst resource scarcity. This situation is further aggravated by increased domestic violence and limited social mobility and safety nets outside the home. Anwar et al., underscore that these multifaceted challenges culminate in a heightened psychological toll on women, as they navigate the intricate web of climate impacts, social norms, and ineffective institutional responses.^27^

Efforts must be concentrated on dismantling and reshaping the conventional gender norms that typify women as a uniform, vulnerable cohort and consider gender equality exclusively a concern for women. It is vital that policies recognize and appreciate the varied experiences and contributions of women in climate initiatives, steering clear of oversimplifying the attributes of both women and men.^26^ Ensuring women’s active participation throughout the stages of climate policy development and execution is paramount. Their unique insights and experiences are invaluable in crafting robust, sustainable, and fair strategies for climate action.^2^ Furthermore, it is imperative to endorse and facilitate women’s leadership in environmental governance. Policies should empower women to assume decision-making roles and lead in climate-related matters, thereby challenging the entrenched gender norms and stereotypes.^28^ Tailoring education and training programs to enhance women’s technical acumen and understanding in climate resilience is another crucial step. Such initiatives can augment their capacity to adapt and position them as pivotal agents of change in this arena.^25^ Moreover, it is essential to recognize and address the intricate interplay between gender and other social factors like race, class, and ethnicity, ensuring that policies cater to the varied necessities of women across different socio-economic landscapes.^29^ Bolstering networks and organizations dedicated to women and focusing on climate resilience can act as conduits for exchanging knowledge and best practices in the fields of climate adaptation and mitigation.^30^ In addition, it is critical to embed gender perspectives comprehensively within all climate change policies and initiatives. This entails performing gender impact evaluations for climate projects and ensuring the incorporation of gender considerations within the mechanisms of climate financing.^31^ These recommendations underscore the significance of confronting gender disparities in climate change mitigation and adaptation, emphasizing that it is not solely a question of fairness but also of effectiveness. Policies should transcend reductive views about women’s roles and ardently work toward empowering them as essential contributors to climate resilience. Such an approach promises not just a more robust response to the climate crisis but also significant strides towards achieving gender equality.

### Policy integration

This theme focuses on the critical analysis of existing policies and the proposal of a comprehensive framework that integrates gender perspectives into climate adaptation and mitigation efforts. Current policies often overlook or inadequately address women’s specific needs, leading to ineffective and non-inclusive outcomes.^5,7^ Intersectional policy analysis helps identify gaps and develop targeted interventions that consider the intersecting identities and experiences of women.^13^

#### Adaptation and resilience policy

Our analysis shows that socio-cultural norms continue to disproportionately heighten women’s vulnerabilities to climate change. Factors such as deprivation of property rights, educational barriers, early marriage, the dowry system, and domestic violence collectively restrict women’s mobility and financial autonomy, making them more susceptible to the adverse impacts of climate stress. These social norms limit women’s access to resources and their ability to respond effectively to climate-induced challenges. Collectively these factors make women more susceptible to the adverse impacts of climate change.^32^ Empirical evidence highlights the importance of addressing these socio-cultural constraints in climate adaptation strategies, as failure to do so perpetuates the structural inequalities that exacerbate women’s vulnerabilities.^32,33^

Furthermore, gender-sensitive vulnerability frameworks are essential in capturing the multi-dimensional aspects of vulnerability, including not just physical exposure to climate risks but also emotional and psychological responses such as fear, anger, and uncertainty.^34^ These frameworks reveal significant differences in how men and women respond to climate stressors, with women often facing additional barriers due to entrenched gender roles and power dynamics in local communities.^35^ These findings underscore the importance of redefining resilience through a gender-sensitive lens to ensure that coping mechanisms address the fundamental causes of vulnerability rather than just surface-level adaptations.

#### Implications for climate adaptation and resilience strategies

Based on these findings, it is clear that policies must be meticulously crafted to deconstruct and reshape the societal norms that marginalize women, particularly in LMICs. Addressing socio-cultural barriers such as property rights deprivation and educational inequality is crucial to fostering a more equitable and resilient society. This “transformation as liberation” ethos requires systemic changes, advocating for a shift in social-technological frameworks to mitigate loss and damage in ways that promote social justice and sustainability.^33^ Such an approach demands active engagement at all levels of society, including global governance, which must prioritize gender equity as a key objective in climate resilience efforts.^1,33^

In terms of policy formulation, it is imperative to integrate women’s insights and experiences into the design of climate change adaptation and mitigation strategies.^2^ Women bring unique perspectives that can significantly enhance the inclusivity and effectiveness of climate policies, particularly when they are actively involved in decision-making processes. The development of gender-transformative policies is essential, as these go beyond gender awareness to reform the structural inequalities that perpetuate women’s vulnerabilities.^36^

Empowerment and capacity-building initiatives play a crucial role in this transformative process. By providing education, training, and leadership opportunities tailored for women, these initiatives aim to enhance women’s decision-making capabilities, leadership skills, and active participation in climate-related activities. Such empowerment is pivotal in dismantling the barriers imposed by entrenched gender roles. Furthermore, the integration of gender considerations into all climate change policies and actions is essential. This necessitates a collaborative and intersectoral approach, involving sectors such as health, education, agriculture, and disaster risk management, to ensure a comprehensive and nuanced response to women’s specific needs and perspectives.

Community engagement and participation are also key in developing and implementing effective climate resilience strategies. Community-based approaches should be inclusive and participatory, ensuring that women’s voices are not just heard but are central in shaping the responses to climate challenges. Finally, the establishment of robust monitoring and evaluation mechanisms is critical to assess the effectiveness of policies aimed at reducing women’s vulnerabilities to climate stress. These mechanisms will help identify gaps, facilitate necessary adjustments, and ensure that the policies meet their intended goals and truly enhance women’s resilience to climate change.

## Discussion

The findings from this analysis highlight the critical need for a multi-faceted approach to enhancing women’s resilience to climate change. Addressing health impacts requires a holistic strategy focused on strengthening health systems and infrastructure, particularly for vulnerable groups such as pregnant women and newborns. Empowerment and capacity building are essential for fostering women’s leadership and participation in climate-related decision-making processes. Moreover, the integration of gender perspectives into climate policies is imperative to ensure that these policies are inclusive and effective. Transformative policies that dismantle socio-cultural barriers and promote gender equality are crucial for creating resilient communities. By harnessing women’s unique insights and experiences, these policies can significantly elevate the effectiveness of climate adaptation and mitigation strategies, ultimately contributing to a more robust response to the climate crisis and achieving gender equality.

The health impacts of climate change on women are profound and multifaceted. As highlighted in the introduction, climate-related stressors such as heatwaves, flooding, and vector-borne diseases disproportionately affect women, particularly pregnant women and newborns.^2,6^ The findings reveal that women in LMICs face higher risks due to inadequate healthcare infrastructure, socio-economic constraints, and cultural barriers.^15,37^ These health disparities necessitate a comprehensive approach to strengthening health systems and infrastructure, ensuring the availability of clean water, sufficient nutrition, and secure shelter during severe climate events.^18,19^

The concept of limbo,^9^ as discussed in this paper, is particularly relevant to understanding the psychological dimensions of women’s experiences with climate change in LMICs. “Limbo” is often experienced by women facing continuous climate-induced adversities, such as displacement, resource scarcity, and disrupted livelihoods. This state of being is marked by heightened anxiety, stress, and a sense of helplessness, exacerbated by inadequate mental health support and socio-economic constraints. Addressing this psychological impact requires targeted mental health interventions that not only provide immediate relief but also build long-term resilience against recurring climate stressors. Integrating mental health services into existing climate adaptation strategies, particularly in community health programs, could help mitigate these adverse effects and improve the overall well-being of women in these vulnerable contexts.

Empowerment and capacity building are essential components of enhancing women's resilience to climate change. Increasing digital health literacy, fostering women’s leadership in climate-related decision-making, and supporting community initiatives are critical strategies.^3,4^ The analysis highlights the importance of intersectional approaches that address the specific needs of women from diverse backgrounds, ensuring that empowerment efforts are inclusive.^25^ This is particularly important in LMICs, where socio-cultural norms often limit women’s access to education and economic opportunities, further exacerbating their vulnerability to climate change.^7^

### Policy integration

The integration of gender perspectives into climate policies is imperative for effective and inclusive climate resilience strategies. The findings indicate that current policies often overlook or inadequately address women’s specific needs, leading to ineffective and non-inclusive outcomes.^5,7^ Intersectional policy analysis helps identify gaps and develop targeted interventions that consider the intersecting identities and experiences of women.^13^ The study emphasizes the need for comprehensive policy frameworks that dismantle socio-cultural barriers, promote gender equality, and enhance women’s capacities to contribute to sustainable solutions.

Based on the literature reviewed and our extensive discussions with experts from the Global North and South here are some concrete challenges and policy solutions that underscore the importance of empowering women through digital health literacy, enhancing their political representation, and strengthening healthcare systems tailored to their specific needs. Furthermore, they highlight the significance of community-based interventions, the protection of sexual and reproductive health rights, the fostering of international collaborations for tailored health services, and the promotion of women’s active involvement in climate decision-making. Collectively, these policy solutions offer a holistic approach to not only mitigate the adverse effects of climate change on women but also to empower them as pivotal agents of change and resilience in the face of escalating environmental challenges.

#### Challenges

The challenges posed by climate change have a profound and multifaceted impact on societies worldwide but more specifically in LMICs. However, not all segments of society bear the brunt of these challenges equally. Women, in particular, find themselves at a nexus of vulnerability, their plight exacerbated by a constellation of socio-economic, health, and policy-related factors. Based on the literature mentioned above, the following challenges and policy solutions are critical that underpin the disproportionate impact of climate change on women:

• Women in LMICs face unique vulnerabilities due to climate-induced environmental changes, which exacerbate their health, socioeconomic, and cultural challenges.
• Prevalent gender biases and societal norms hinder women’s access to resources and opportunities, limiting their capacity to respond to climate challenges effectively.
• Climate change poses severe risks to pregnant women and newborns, demanding urgent attention to healthcare systems and services.
• Current climate resilience and adaptation strategies often overlook the critical role of gender considerations, leading to ineffective and non-inclusive policies.
• The underrepresentation of women in political decision-making processes restricts their influence on climate-related policies and actions.
• A lack of emphasis on enhancing women’s digital health literacy restricts their ability to access crucial information and services related to climate resilience and health.
• Climate-induced disasters often disrupt access to essential sexual and reproductive health services, disproportionately affecting women in vulnerable regions.
• Women’s insights and experiences are often underutilized in climate resilience and sustainability efforts, limiting the effectiveness of these initiatives.

**Figure 1.**
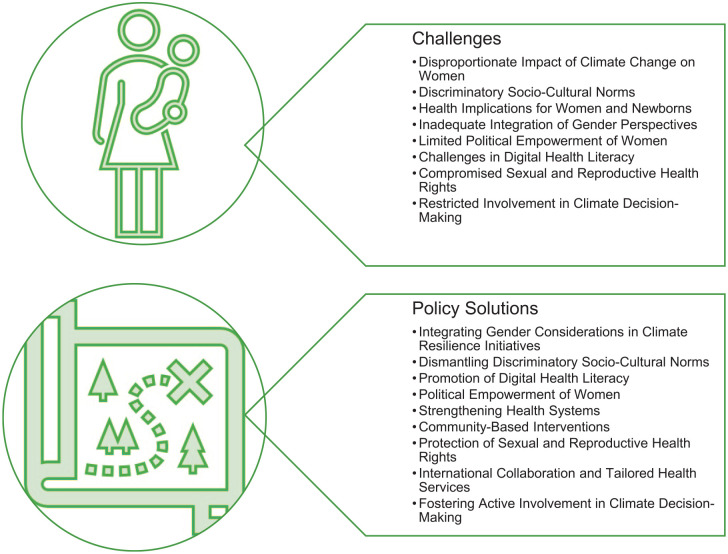
Strategies for enhancing women’s resilience to climate change.

These issues identify the problems that not only restrict the involvement of women in climate decision-making processes but also exacerbate their vulnerability to climate change. These intertwined issues not only underscore the unique vulnerabilities that women face in the wake of climate change but also call for an urgent re-evaluation of current strategies to ensure inclusivity, equity, and effectiveness in climate resilience initiatives. See Figure 1 for challenges and policy solutions.

#### Policy solutions and example case studies

To counteract the disproportionate impact of climate change on women in LMICs, it is vital to transition from mere problem identification to the actualization of robust policy solutions. This section introduces a comprehensive suite of policy recommendations, each meticulously designed to address the multifaceted challenges faced by women in the context of climate change. From integrating gender considerations in climate resilience initiatives to dismantling deeply entrenched discriminatory socio-cultural norms, these policies aim to create a transformative shift in how women’s needs and strengths are perceived and prioritized in climate adaptation and mitigation strategies.

• Policies should systematically incorporate gender perspectives to ensure that the unique needs and strengths of women are recognized and addressed in climate adaptation and mitigation strategies. An example case study for this can be women community groups in Lahore, Pakistan who tackled the solid waste pollution problem by mapping local resources and vulnerabilities and developing partnerships with the local government. They established waste management task forces and installed trashcans, transforming waste management in their community. More than 11,000 women benefited, leading campaigns, and creating awareness about climate change. They developed a vulnerability index tool to assess risks and proposed cost-effective climate change kits for emergencies. This case study showcases how women can have an important role to play in local climate adaptation efforts.^38^ Advocating for social change to eradicate gender biases, thereby enhancing women’s capacities and rights in the face of climate challenges. In India, the Self-Employed Women’s Association (SEWA) has played a crucial role in advocating for social change to dismantle gender biases. SEWAs initiatives include legal aid, microfinance, and capacity-building programs that empower women economically and socially, thereby enhancing their resilience to climate impacts.^39^
• Empowering women by improving their digital health literacy, enabling them to access vital information and services related to climate resilience and health. In a case study from Pakistan, it was highlighted the impacts of climate-induced migration on women are more disproportionate. In this study conducted in Muzaffargarh and Tharparkar districts, it was shown that women face significant hardships during migration, including health impacts, loss of unborn children, harassment, and gender-based violence. The study underscores the need for improved education, water availability, safe shelters, road connectivity, healthcare services, and migration management to enhance climate adaptation for local communities.^40^ The authors suggested the need for more awareness and digital literacy as a crucial solution.
• Encouraging and facilitating the increased participation of women in political discourse and decision-making processes, especially concerning climate policy. The Women’s Environment and Development Organization supports the “Women Delegates Fund,” which increases women’s participation in international climate negotiations. According to UNFCCC, This initiative has enhanced the presence of women in political discourse and decision-making processes related to climate policy.^41^
• Adopting a multi-sectoral approach to fortify health services, particularly focusing on the needs of pregnant women and newborns amidst climate crises. Similarly, Lovell et al. successfully integrated gender considerations The BRACED program, managed by the Overseas Development Institute, used intersectional approaches in Kenya and Nepal to integrate gender considerations into climate resilience initiatives. In Kenya, the study focused on how gender, ethnicity, literacy, and political representation influence resilience to natural hazards. The findings underscored the importance of recognizing intersecting inequalities to strengthen resilience effectively.^42^ Encouraging localized, community-driven initiatives to address and mitigate the health impacts of climate change on women. In Vhembe District, South Africa, women engage in subsistence farming and are particularly vulnerable to the impacts of climate change. Local women have adopted climate-resilient agricultural practices, which not only help manage the adverse effects of climate variability but also improve their food security and health. The community participates in knowledge-sharing and capacity-building workshops which enhance their resilience and adaptability to climate stressors. This example illustrates how empowering women with the right skills and knowledge can lead to sustainable community development and improved health outcomes amidst climate challenges.^43^
• Implementing a comprehensive reproductive justice framework to ensure uninterrupted access to critical sexual and reproductive health services, especially during climate-induced disasters. In a case study, conducted by ActionAid, UNHCR, and CARE Bangladesh, provides an intersectional analysis of gender among Rohingya refugees and host communities in Cox’s Bazar, Bangladesh. It highlights the compounded vulnerabilities of women and girls, especially those with disabilities, due to socio-cultural and gender norms, and suggests tailored humanitarian interventions based on periodic age, gender, and diversity analysis.^44^
• Promoting global partnerships and advocating for customized health services in LMICs to address the specific health challenges posed by climate change. Intersectional approaches to forests have highlighted the gender-differentiated knowledge of forest flora and fauna between both inter-categorical (e.g., women and men, girls and boys, and non-binary people) and intra-categorical gender intersections (e.g., indigenous and non-indigenous women, younger and elder women). For instance, indigenous elder women often serve as guardians of traditional knowledge regarding forest flora and fauna, illustrating the importance of intra-categorical approaches.^45^
• Ensuring that women are not just beneficiaries but active contributors and leaders in formulating and implementing climate resilience strategies. In Nepal, projects focusing on water governance have emphasized the critical role of women in managing and conserving water resources.^46^ Women are often primary water collectors and managers, and their participation in decision-making processes has been crucial for effective water governance.

These policy solutions underscore the importance of empowering women through digital health literacy, enhancing their political representation, and strengthening healthcare systems tailored to their specific needs. Furthermore, it highlights the significance of community-based interventions, the protection of sexual and reproductive health rights, the fostering of international collaborations for tailored health services, and the promotion of women’s active involvement in climate decision-making. Collectively, these policy solutions offer a holistic approach to not only mitigate the adverse effects of climate change on women but also to empower them as pivotal agents of change and resilience in the face of escalating environmental challenges.

In addressing women’s vulnerabilities necessitates a transformative approach that extends beyond the boundaries of traditional resilience frameworks. It’s crucial to undertake a comprehensive reassessment and restructuring of socio-cultural norms, ensuring women are not only empowered but also have their needs and perspectives thoroughly integrated into all areas of policy formulation. This multifaceted strategy is paramount, not just for reinforcing women’s resilience, but also for enhancing the overall robustness, sustainability, and effectiveness of climate change mitigation and adaptation initiatives.^47^

By bolstering women’s awareness and involvement and ensuring digital health literacy, it is possible to empower them to take an active role in addressing the multifarious health impacts of climate change. This empowerment is not limited to individual resilience; it catalyzes collective action and societal transformation in confronting climate-related challenges. Enhancing women’s digital literacy equips them with the necessary tools and insights to navigate the complexities of climate change and its health implications, thereby fostering a more informed and proactive stance in climate action.^48^

Furthermore, the intersection of women’s political empowerment and child health in LMICs presents compelling evidence of the broader societal benefits of female participation in political processes. Studies demonstrate a positive correlation between women’s political empowerment and improved health outcomes, such as better nutrition and higher immunization rates among children. This correlation underscores the imperative for increased female participation in political discourse and decision-making, especially in the context of climate change. As climate change poses increasingly severe threats to health systems and societal well-being, incorporating women’s perspectives in political decision-making is not just beneficial; it’s essential for fostering resilient, health-conscious governance and policymaking.^49^ Moreover, the escalating challenges posed by climate change on sexual and reproductive health rights underscore the urgency of adopting a comprehensive reproductive justice framework. In the face of climate-induced disasters, access to critical sexual and reproductive health services is often compromised, exacerbating vulnerabilities, particularly for women in affected regions. A reproductive justice framework ensures that these essential services are safeguarded and that the rights and needs of women are prioritized and adequately addressed, even in the most challenging and unpredictable climate scenarios.^50^

While this paper provides a comprehensive framework for enhancing women’s resilience to climate change in LMICs, limitations specific to the scope and methodology of this study must be acknowledged. Firstly, the reliance on secondary sources and a qualitative narrative synthesis approach means that the conclusions drawn are primarily based on existing literature and expert opinions. This approach may not fully capture the rapidly changing dynamics of climate change impacts on women’s health, particularly in diverse and localized contexts within LMICs. Future studies could benefit from primary data collection that includes lived experiences and voices directly from the communities of women most affected by climate change to provide more context-specific insights and validation of the proposed strategies.

Additionally, the paper emphasizes policy recommendations and transformative strategies, but these may lack the granularity needed for application in different socio-cultural and political contexts within LMICs. For example, while advocating for digital health literacy and political empowerment, the paper does not address the varying levels of digital access and political engagement among women across different LMIC regions. The effectiveness of these strategies can differ greatly depending on local technological infrastructure, cultural norms, and governance structures. Future research should focus on tailoring these strategies to specific contexts by conducting in-depth, localized case studies and pilot projects to explore the unique barriers and facilitators to implementing these policy recommendations.

## Conclusion

In summary, a holistic and inclusive approach to climate resilience is paramount. By integrating digital literacy, advocating for women’s political empowerment, and steadfastly upholding reproductive health rights into climate resilience strategies, policies are not only rendered more inclusive and equitable but are also significantly strengthened in their capacity to confront and mitigate the myriad challenges presented by climate change. This approach ensures that women, who are often on the frontlines of climate impact, are not merely beneficiaries of climate policies but are active, empowered participants and leaders in shaping a resilient and sustainable future.

To build resilience in women’s health against climate change, policies must prioritize gender equality, empower women politically and digitally, and adopt a transformational approach to resilience. Addressing the unique challenges faced by women due to climate change requires a multi-faceted strategy that includes enhancing their political representation, improving access to health services, and integrating gender perspectives into climate policies and practices. This holistic approach will not only enhance women’s resilience to climate change but also contribute to achieving broader sustainable development goals.

## Declarations

**Ethics approval and consent to participate:** Not applicable.

**Consent for publication:** Not applicable.

**Availability of data and materials:** Data sharing is not applicable to this article as no datasets were generated or analyzed during the current study.
